# [^18^F]AlF-NOTA-ADH-1: A new PET molecular radiotracer for imaging of N-cadherin-positive tumors

**DOI:** 10.3389/fonc.2023.1126721

**Published:** 2023-05-22

**Authors:** Zhenfeng Liu, Guanghua Wen, Yuqiao Huang, Yanzhao Dong, Zewei Wang, Ahmad Alhaskawi, Shuyi Zhang, GuoLin Wang, Qianni Ye, Haiying Zhou, Hui Lu, Mengjie Dong

**Affiliations:** ^1^ Department of Nuclear Medicine, The First Affiliated Hospital, Zhejiang University School of Medicine, Hangzhou, China; ^2^ Department of Nuclear Medicine, Shenzhen Longhua District Central Hospital, Shenzhen, China; ^3^ Institute of Translational Medicine, Zhejiang University, Hangzhou, China; ^4^ Department of Clinical Medicine, Zhejiang University School of Medicine, Hangzhou, China; ^5^ Department of Orthopedics, The First Affiliated Hospital, Zhejiang University School of Medicine, Hangzhou, China; ^6^ Department of Nuclear Medicine, Peking University Shenzhen Hospital, Shenzhen, China

**Keywords:** PET imaging probe, N-cadherin, [18 F]AlF-NOTA-ADH-1, cancer, EMT

## Abstract

**Background:**

The cell adhesion molecule (CAM) N-cadherin has become an important target for tumor therapy. The N-cadherin antagonist, ADH-1, exerts significant antitumor activity against N-cadherin-expressing cancers.

**Methods:**

In this study, [^18^F]AlF-NOTA-ADH-1 was radiosynthesized. An in vitro cell binding test was performed, and the biodistribution and micro-PET imaging of the probe targeting N-cadherin were also studied in vivo.

**Results:**

Radiolabeling of ADH-1 with [^18^F]AlF achieved a yield of up to 30% (not decay-corrected) with a radiochemical purity of >97%. The cell uptake study showed that Cy3-ADH-1 binds to SW480 cells but weakly binds to BXPC3 cells in the same concentration range. The biodistribution results demonstrated that [^18^F]AlF-NOTA-ADH-1 had a good tumor/muscle ratio (8.70±2.68) in patient-derived xenograft (PDX) tumor xenografts but a lower tumor/muscle ratio (1.91±0.69) in SW480 tumor xenografts and lowest tumor/muscle ratio (0.96±0.32) in BXPC3 tumor xenografts at 1 h post-injection (p.i.) These findings were in accordance with the immunohistochemistry results. The micro PET imaging results revealed good [18F]AlF-NOTA-ADH-1 tumor uptake in pancreatic cancer PDX xenografts with strong positive N-calcium expression, while lower tumor uptake in SW480 xenografts with positive expression of N-cadherin, and significantly lower tumor uptake in BXPC3 xenografts with low expression of N-cadherin, which was consistent with the biodistribution and immunohistochemistry results. The N-cadherin-specific binding of [18F]AlF-NOTA-ADH-1 was further verified by a blocking experiment involving coinjection of a non radiolabeled ADH-1 peptide, resulting in a significant reduction in tumor uptake in PDX xenografts and SW480 tumor.

**Conclusion:**

[^18^F]AlF-NOTA-ADH-1 was successfully radiosynthesized, and Cy3-ADH-1 showed favorable N-cadherin-specific targeting ability by in vitro data. The biodistribution and microPET imaging of the probe further showed that [18F]AlF-NOTA-ADH-1 could discern different expressions of N-cadherin in tumors. Collectively, the findings demonstrated the potential of [^18^F]AlF-NOTA-ADH-1 as a PET imaging probe for non-invasive evaluation of the N-cadherin expression in tumors.

## 1 Introduction

Tumor invasion and metastasis are the key processes in tumor deterioration and are associated with poor prognosis. In recent years, a growing number of studies have found that the metastasis of most tumors depends on the epithelial-mesenchymal transition (EMT) (1–4). EMT is a biological developmental process characterized by epithelial cells losing the epithelial features and acquiring mesenchymal properties (5). The process of EMT is accompanied by epithelial cadherin (E-cadherin) down-regulation and the concomitant up-regulation of neural cadherin (N-cadherin) (6–9). Transfection of N-cadherin into tumor cells with negative N-cadherin expression resulted in significantly higher aggressiveness, while the intercellular adhesion mediated by E-cadherin disappeared, and the expression of E-cadherin was significantly decreased (10, 11). N-cadherin is absent or has low expression in normal epithelial cells, and abnormal expression of N-cadherin has been associated with epithelial malignancies such as breast cancer, prostate cancer, and uroepithelial carcinoma (12, 13). Considering the high expression of N-cadherin in tumors, it can be an excellent target for tumor treatment and diagnosis. Controlling tumor metastasis and reducing drug resistance by inhibiting N-cadherin may be an effective treatment strategy (14, 15). The pentapeptide ADH-1 is an N-cadherin inhibitor (12, 16–19) that specifically binds to N-cadherin through hydrophobic and electrostatic interactions, involving the interaction between the Trp2 residue of the binding site of N-cadherin with the Ala fragment of the ADH-1 molecule (20). Applying ADH-1 to tumors can lead to tumor vascular angiolysis and apoptosis (21–23), but does not damage normal mature blood vessels (22, 23). A combination of ADH-1 and melphalan was explored in the treatment of melanoma in mice, which significantly reduced tumor growth. The effect was equivalent to 30 times the dose of melphalan alone (23). The enhanced response of ADH-1 to melphalan was associated with increased tumor cell apoptosis, increased DNA adduct formation, and altered intracellular signaling. Clinical experiments investigating ADH-1 (phase 1 and phase 2 single-agent studies) showed anti-cancer activity in patients with N-cadherin–positive tumors (17, 24).

As an emerging field, molecular imaging has attracted significant attention and developed quickly in recent years. The combination of small peptides or small molecules with positron nuclide labeling can be used as new molecular probes for functional imaging (25). Developing a molecular probe targeting N-cadherin for noninvasive imaging of N-cadherin *in vivo* has potential application value in tumor diagnosis, drug development, dose optimization, and treatment monitoring. In this study, a novel PET probe [^18^F]AlF-NOTA-ADH-1 was prepared by using ADH-1 peptide, and the feasibility of [^18^F]AlF-NOTA-ADH-1 for the detection of N-cadherin positive tumors was explored.

## 2 Materials and methods

### 2.1 General

The NOTA was purchased from Macrocyclic, Inc; protected amino acids, other peptide synthesis reagents, and resins were obtained from Nanchang tanzhenbio Co., Ltd; anhydrous aluminum chloride (AlCl_3_) and sodium acetate were purchased from Alfa Aesar (China) Chemicals Co., Ltd; and trifluoroacetic acid (TFA) was obtained from Shanghai Aladdin Biochemical Technology Co., Ltd. (China). Reagents, including phosphate-buffered saline (PBS) and cell culture medium, were obtained from Sigma-Aldrich. Fetal bovine serum (FBS) was purchased from Biological Industries (BI) company. All reagents and solvents were commercial products and used without further purification. The Siemens Cyclotron produced [^18^F] fluoride by bombarding a [^18^O] H_2_O target with 12.5 MeV protons. Analytical HPLC (high-performance liquid chromatography) was performed on an Agilent 1200 system with a reversed-phase column (Agilent Zorbax ODS, 250 × 4.6 mm). Mass spectra were obtained on a Q-T of a premier UPLC system equipped with an electrospray interface (ESI) (Waters, USA). A γ-counter (CAPRA-R, Capintec, Inc., Ramsey, New Jersey, USA) was used to measure the radiation value of the biological distribution experiment. Small animal imaging was performed using a micro-PET/CT scanner (Siemens Inveon Multimodality System, Germany).

### 2.2 Synthesis of peptides

**Scheme 1:**
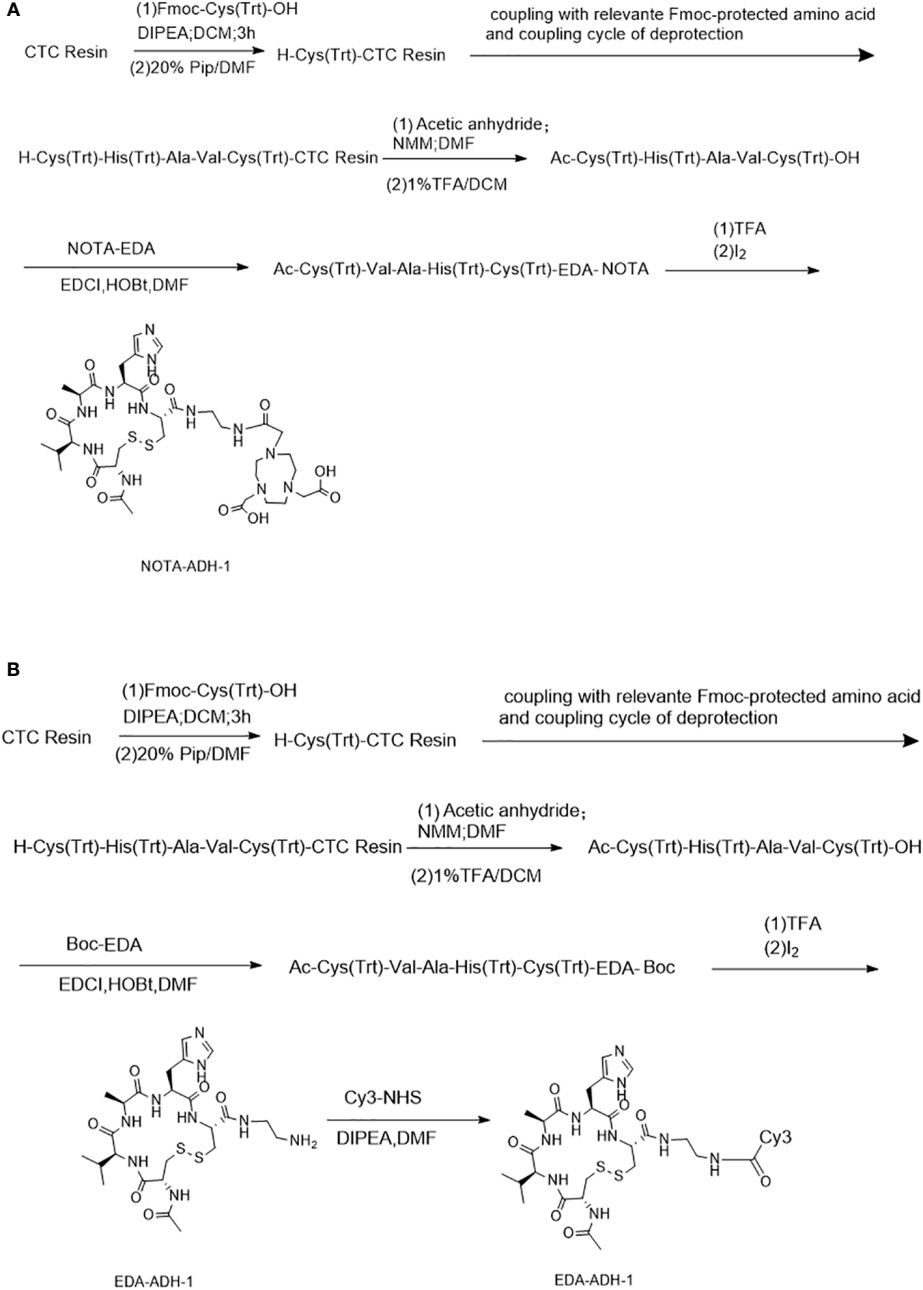
The synthesis process of the peptides of NOTA-ADH-1 **(A)** and Cy3-ADH-1 **(B)**.

**Scheme 1** describes the peptides synthesized by the solid-phase synthesis method (26). The purity of the peptides, including NOTA-ADH-1 and Cy3-ADH-1, was identified by high-performance liquid chromatography (HPLC), and the molecular weight was identified by mass spectrometry. They were stored at -20°C after freeze-drying.

### 2.3 Cell binding studies

Human colorectal carcinoma SW480 and pancreatic BxPC3 cell lines were purchased from the Institute of Biochemistry and Cell Biology, the Shanghai Institute for Biological Sciences, and the Chinese Academy of Sciences. Specific binding studies were evaluated on SW480 and BxPC3 tumor cells. SW480 and BxPC3 cells were cultured in a medium containing high glucose and supplemented with fetal bovine serum, glutamine, and penicillin-streptomycin, and the culture medium was changed every other day. Cells were expanded in tissue culture pans in humidified air containing 5% carbon dioxide at 37°C and detached with trypsin and phosphate-buffered saline for further cell culture when cells were confluent.

To assess the binding ability of the peptide, the Cy3-ADH-1 peptide was synthesized. An equal number (1×10^5^) of SW480 and BxPC3 cells were seeded in 6-well plates. After overnight incubation, 0.5 ml of culture medium and 0.5 ml of Cy3-ADH-1 solution with concentrations 5 μM, 20 μM, 50 μM, 100 μM, and 200 μM were added to the cell chambers in the dark, respectively, and incubated at 37°C in a 5% CO_2_ incubator for half an hour. Subsequently, the solution in the 6-well plates was discarded, and the cells were fixed with paraformaldehyde for 10 min. After washing with PBS three times, the cells were mounted in a mounting medium containing DAPI for 15 minutes and then visualized by an Olympus IX71 fluorescence microscope (Olympus, Japan).

A competitive binding assay was performed on SW480 tumor cells. Therefore, 1×10^5^ SW480 cells were placed in 3-well plates, and 0.5 ml of unlabeled ADH-1 solution of 0 μM, 50 μM, and 200 μM concentration were added to each well and incubated at 37°C in binding buffer. After 30 minutes of reaction, 0.5 ml of 10 μM Cy3-ADH-1 solution was added and then incubated in binding buffer at 37°C for another 1 hour to allow fluorescently-labeled polypeptides and excess non-labeled polypeptides to combine with tumor cells. The cells were then washed 3 times with 1ml PBS solution for 5 min each time and were mounted in a mounting medium containing DAPI for 15 minutes. The uptake intensity of SW480 colon cancer cells to fluorescently-labeled polypeptides was observed and measured by a fluorescence microscope.

### 2.4 Preparation of [^18^F]AlF-NOTA-ADH-1 

**Scheme 2:**
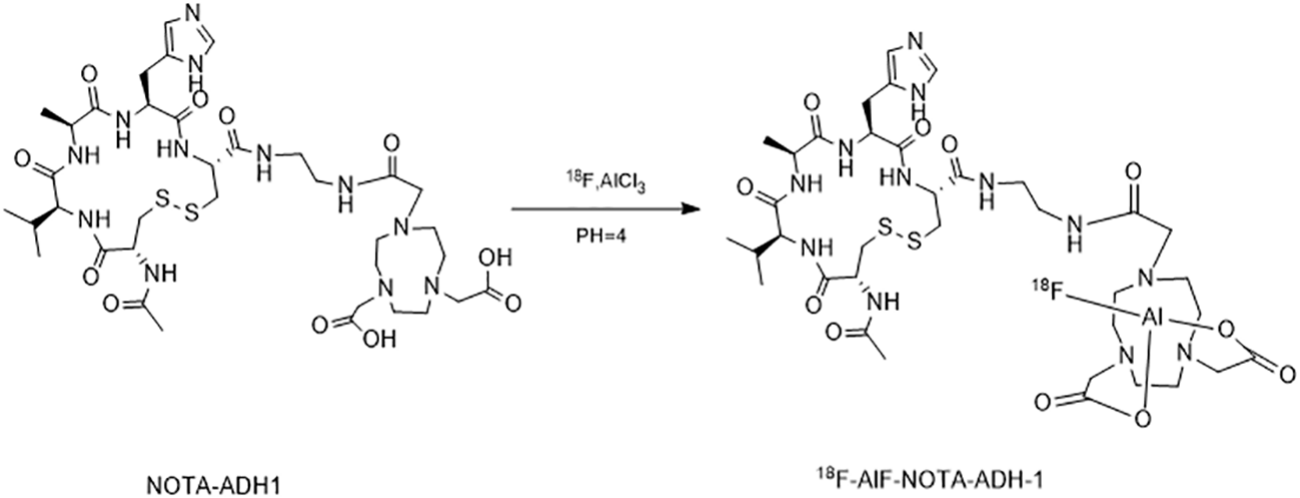
The radiosynthesis routine of ^18^F -AlF-NOTA-ADH-1.

**Scheme 2** describes the radiosynthesis routine of [^18^F]AlF-NOTA-ADH-1 (27–31). 100ug of NOTA-ADH-1 was dissolved in 2ml axygen in a centrifuge tube with 400ul pure water; AlCl_3_ (26.0 nmol; 13.0 μL; 2.0 mM) in sodium acetate buffer solution (pH 4; 0.2 M) and 1mL acetonitrile was added to the reactor and mixed well. 500μl ^18^F target water (1000-1500mCi) was added to the reactor and heated at 100°C for 10min; 5uL of crude production point samples were collected for Thin-layer chromatography (TLC) detection. The remaining crude product was slightly cooled and then transferred to the pre-conditioned HLB cartridge with 15ml water and washed with 10ml PBS and 20ml water, respectively. The product was finally eluted with 2.0 mL of ethanol and water at a ratio of 1:1 (V/V), filtered using a sterile f**i**ltration membrane, and collected in the receiving bottle. The product (310-500mCi) was then diluted into a solution containing 5% ethanol using normal saline for injection, and samples were taken for quality control.

### 2.5 Determination of radiochemical purity

The radiochemical yield was monitored by TLC (50% acetonitrile), and the radiochemical purity was confirmed by HPLC using a Zorbax ODS(C18) 4.6 * 250 mm analysis column. The HPLC included the mobile phase A, a 0.1% acetonitrile solution of trifluoroacetic acid (TFA), and phase B, an aqueous solution of 0.1% TFA. The mobile phases were performed to carry out a gradient elution. The ratio of phase A to phase B at 0 min was 10%:90% and gradually rose to 70%:30% at 20 min, which was maintained at a flow rate of 1mL/min.

### 2.6 *In vitro* stability determination

The stability of [^18^F]AlF-NOTA-ADH-1 was tested in PBS and bovine serum. In brief, 3.7 MBq of[^18^F]AlF-NOTA-ADH-1 was pipetted into 0.5 mL of PBS and incubated in PBS at room temperature or bovine serum at 37°C. For the study, an aliquot of the solution was directly taken at 1, 6, and 8 h after incubation, and the radiochemical purity was determined by radio-TLC or radio-HPLC under identical conditions.

### 2.7 Lipid-water partition coefficient

The partition coefficient of [^18^F]AlF-NOTA-ADH-1 was measured by assessing the distribution of radioactivity in 1-octanol and phosphate buffer in a 2 mL centrifuge tube. 20µL of [^18^F]AlF-NOTA-ADH-1 solution was added to a tube containing 0.5 mL each of 1-octanol and PBS. The mixture was vortexed and centrifuged (5,000 r/min) for 5 min. A total of 2 samples (50 μL) were collected from each layer and were assayed in a γ counter. Partition coefficients (log Po/w) are shown as the log counts in 1-octanol vs. PBS layers (n=3).

### 2.8 Tumor implantation in mice

All animal experiments were performed in accordance with and approved by the Institutional Animal Care and Use Committee guidelines of the First Affiliated Hospital of Zhejiang University School of Medicine. BALB/c nu/nu mice (female, 20 ± 3 g, 4- to 6-week-old; Department of Laboratory Animal Science, Zhejiang University) were used in this study. The SW480 and BxPC3 xenografts were generated, and 5×10^6^ tumor cells were subcutaneously injected into the flanks of female athymic nude mice. The PDX-bearing mouse models were established as follows. Pancreatic cancer (diagnosed as pancreatic ductal adenocarcinoma) tissues were obtained from the surgery of a 67-year-old female patient. Written informed consent was obtained from the patient, and the research protocol was approved by the Clinical Research Ethics Committee of the First Affiliated Hospital of Zhejiang University School of Medicine. The sample was sectioned into about 1mm^3^ pieces, and the tumor tissues were subcutaneously implanted into BALB/c nu/nu mice flanks. Mice were allowed free access to sterile water and food and were kept under strict disease-free conditions with controlled temperature (~25°C), humidity (50-70%), and circadian rhythms (12-hour light/dark cycles). All necessary procedures were carried out to minimize discomfort and avoid the waste of animals. The length and width of the tumor were measured every other day. Biodistribution studies and positron emission tomography in mice were performed after the tumor volume reached 150 to 200 mm^3^.

### 2.9 Biodistribution in normal mice

Thirty SPF Kunming mice weighing 18-20g were randomly divided into 6 groups (n=5). As described above, [^18^F]AlF-NOTA-ADH-1 was purified and separated by HPLC analysis. Each mouse was injected with a dose of 0.74MBq (20μCi) through a caudal vein. Blood was collected from the orbit, the mice were sacrificed, and the brain, heart, lung, liver, spleen, kidney, stomach, intestine, muscle, bone and other organs were taken at 2 min, 5 min, 15 min, 30 min, 60min and 120min after injection. The radioactivity results were recorded as injected radioactivity per gram of tissue (% ID/g) corrected for background and decay.

### 2.10 Biodistribution in the tumor xenograft model

The BALB/c nude mice were randomized into different tumor models (SW480, BxPC3, and pancreatic PDX), including 5 mice per group. Each nude mouse was injected with 0.74MBq (20μCi) [^18^F]AlF-NOTA-ADH-1 through the caudal vein. Blood was collected from the orbit, and the mice were dissected to collect the brain, heart, lung, liver, liver, spleen, stomach, intestine, muscle, bone and others at 30 min, 30min, 60min, 90min and 120min after injection. In the blocked group, the study was performed with a saturating dose of ADH-1 (20 mg/kg of mouse body weight) intravenously administered 60min before the intravenous injection of [^18^F]AlF-NOTA-ADH-1 in the pancreatic PDX model and SW480 xenograft model. The mice in the tumor xenograft model were sacrificed 1h after [^18^F]AlF-NOTA-ADH-1 injection. The nude mice were dissected, and tumor tissues and related tissues (blood, brain, heart, lung, liver, spleen, kidney, stomach, intestine, muscle and bone) were weighed, and their radioactivity was measured by γ-counter. The radioactivity results were recorded as % ID/g.

### 2.11 Micro-PET imaging

[^18^F]AlF-NOTA-ADH-1 was evaluated by micro-PET imaging on nude mice bearing a PDX tumor, SW-480 and BxPC3 tumor xenografts. Each tumor-bearing nude mouse was injected with 3.7MBq [^18^F]AlF-NOTA-ADH-1 *via* the tail vein. After being anesthetized with isoflurane, tumor-bearing nude mice were imaged using a micro-PET/CT scanner at 60 minutes post-injection under continuous isoflurane inhalation using a nasal mask with a connecting tube. Micro-PET/CT scanning was performed after low-dose 3D acquisition CT scanning at 10 minutes per bed. A blocking study was conducted, in which a saturating dose of unlabeled ADH-1 (20 mg/kg of mouse body weight) was intravenously administered 60 min before the intravenous injection of [^18^F]AlF-NOTA-ADH-1.

The three-dimensional volume of interest (VOI) was used to assess the standard uptake value (SUV) of selected organs. The Seimens Inveon analysis software configured with micro-PET/CT was used to delineate the region of interest along the edge of the tumor and each normal tissue on the PET image. Subsequently, the SUV of each region of interest was measured, and the tumor/non-tumor ratio (T/NT) between the tumor and normal tissue was measured.

### 2.12 Immunohistostaining

Tumor tissues were fixed in 10% methanol for 4 hours. The samples were then dehydrated in graded ethanol, embedded in paraffin, and sliced into 5 μm sections. Sections were incubated with N-cadherin (mouse monoclonal antibody, 1:200, Abcam) and then goat anti-mouse secondary antibodies (Abcam diluted 1:200). The secondary antibodies were formed by binding horseradish peroxidase (HRP) and Goat anti-mouse IgG. Then, the sections were stained with diaminobenzidine (DAB )staining solution. DAB generates brown precipitates under the catalysis of HRP, thereby amplifying signals and developing colors. After a series of routine processing, immunohistochemical images were finally obtained.

### 2.13 Statistical analysis

SPSS 26.0 software was used for analysis, and all quantitative data in the experiment were expressed as mean ± SD. *t*-test analysis was used for the comparison of differences between two groups; analysis of variance was used for comparison between multiple organizations. A *p-*value less than 0.05 was considered statistically significant.

## 3 Results

### 3.1 Synthesis of peptides

A standard FMOC solid-phase synthesis was used to synthesize NOTA-ADH-1 and Cy3-ADH-1 (**Scheme 1**). The synthesized peptide was characterized by High Resolution Mass Spectrometry (HRMS) (**Supplementary Figures S1**, **S2**), and its purity was determined by HPLC, revealing a chemical purity of greater than 95%.

### 3.2 *In vitro* cell binding test

**Figure 1:**
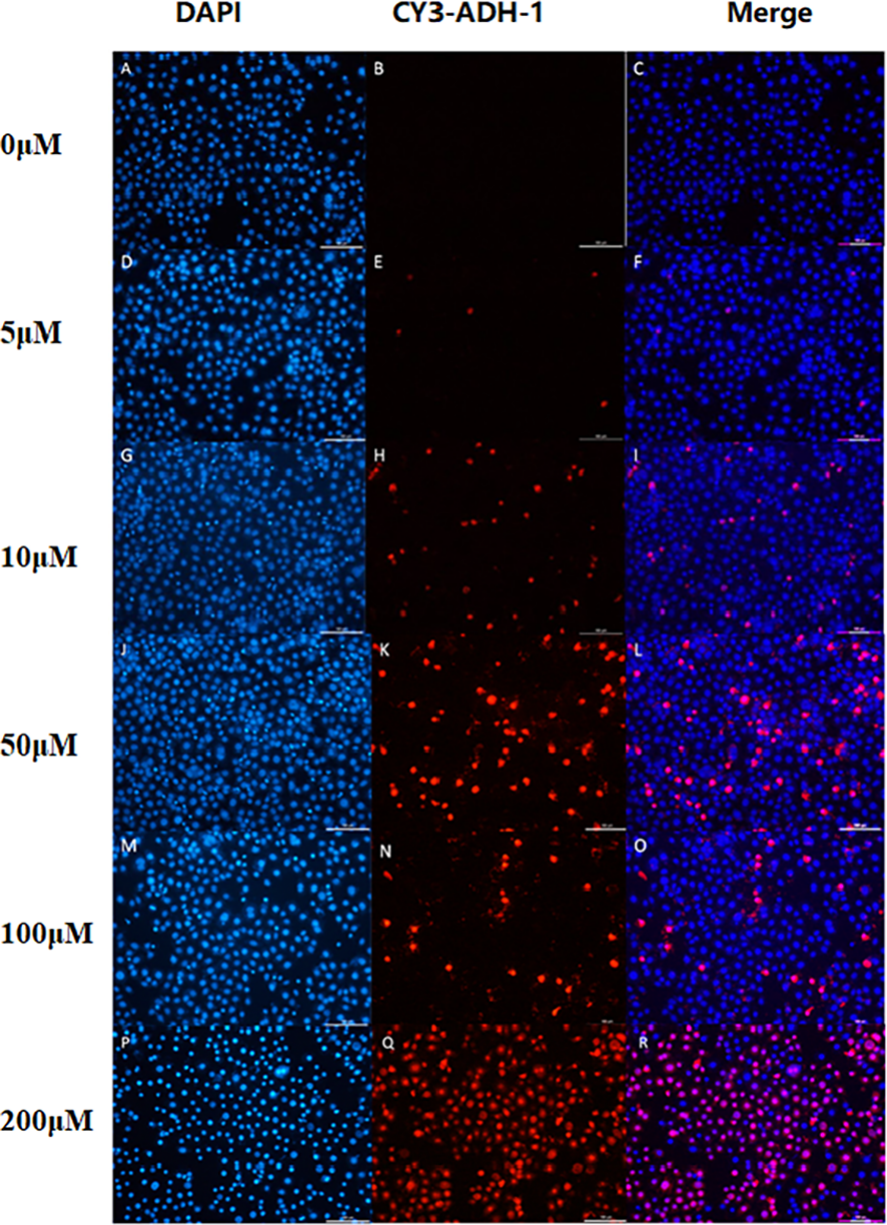
Cell binding assay showed the effect of Cy3-ADH-1 at different concentrations on colorectal cancer SW480 cells. 0μM **(A–C)**,5μM **(D–F)**, 10μM **(G–I)**, 50μM **(J–L)**, 100μM**(M–O)**, 200μM**(P–R)**.

**Figure 2:**
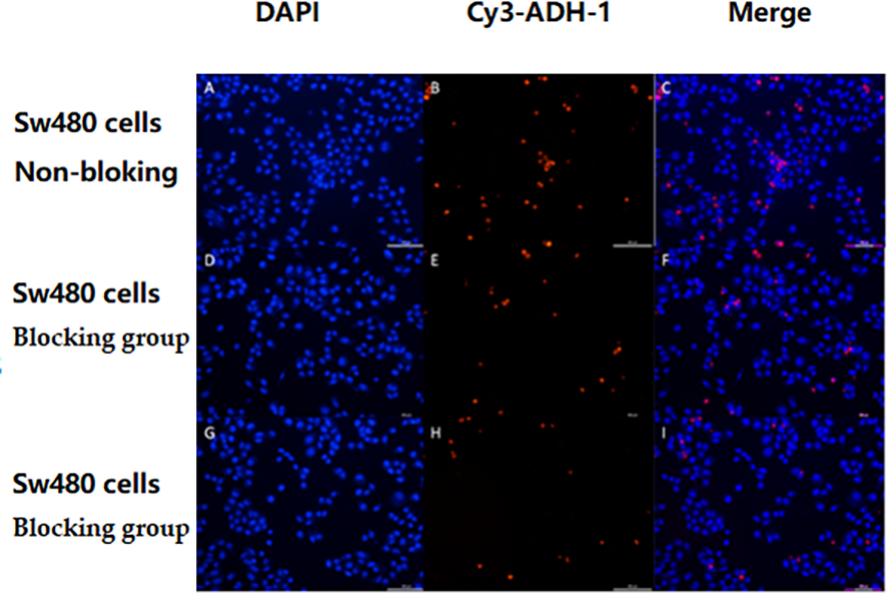
SW480 cell competition inhibition test showed that non-labeled ADH-1 reduced levels of the cellular uptake of Cy3-ADH-1. No-Blocked **(A–C)**, Blocked group at a concentration of 50nM **(D–F)**, Blocked group at a concentration of 200 nM **(G–I)**.

**Figure 3:**
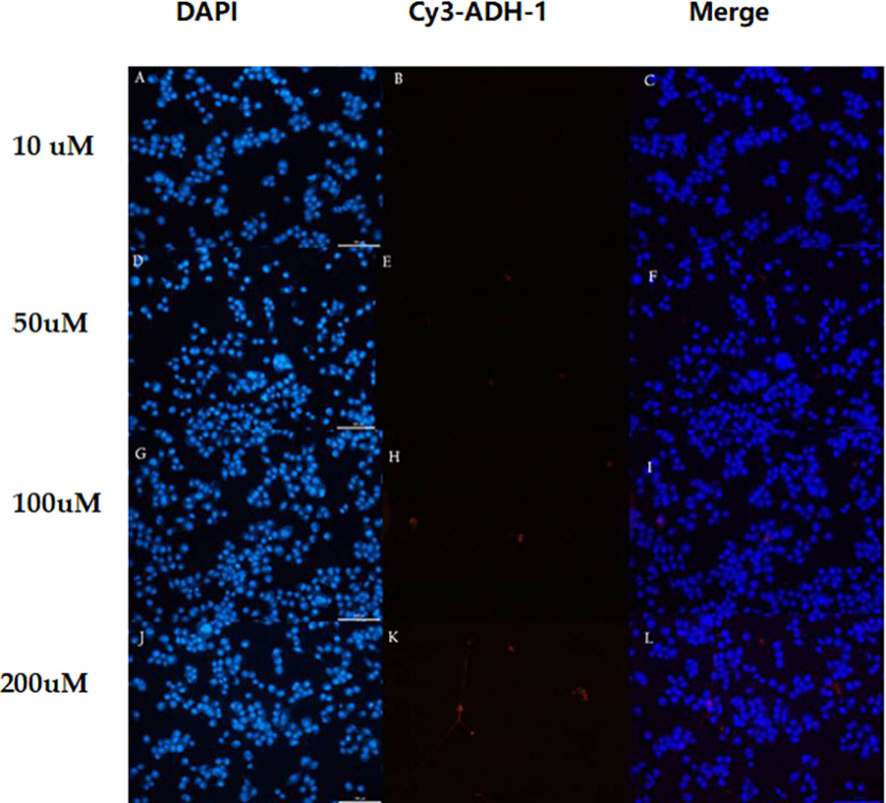
In different concentrations of Cy3-ADH-1, BxPC3 cells showed no obvious fluorescence signal. 10 uM **(A–C)**,50uM **(D–F)**,100uM **(G–I)** and 200uM **(J–L)**.

The *in vitro* results showed that the degree of SW480 cell uptake of Cy3-ADH-1 was concentration-dependent, exhibiting an increasing fluorescence intensity with increasing Cy3-ADH-1 concentration. In contrast, no obvious fluorescence signal was found in the blank control tube (**Figure 1**). Competitive inhibition assays demonstrated that unlabeled ADH-1 significantly affected the uptake of Cy3-ADH-1 in SW480 cells, with the 200nM inhibition group showing a substantially higher uptake than the 50nM inhibition group (**Figure 2**). No significant uptake (or fluorescence signal) of Cy3-ADH-1 by BXPC3 cells was observed in the concentration range of 5 μM to 200 μM (**Figure 3**).

### 3.3 Radiochemical synthesis

**Figure 4:**
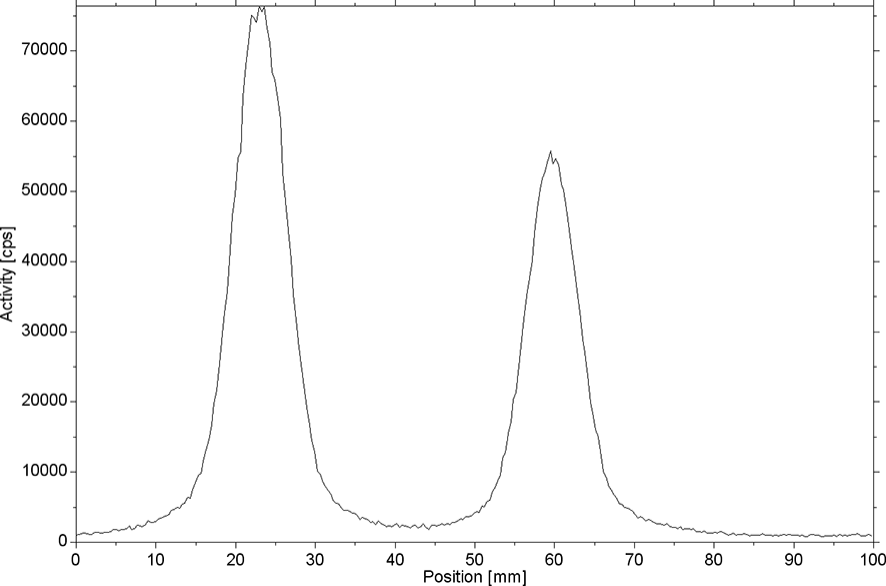
The analytical chromatograms from radio TLC of the crude product.

**Figure 5:**
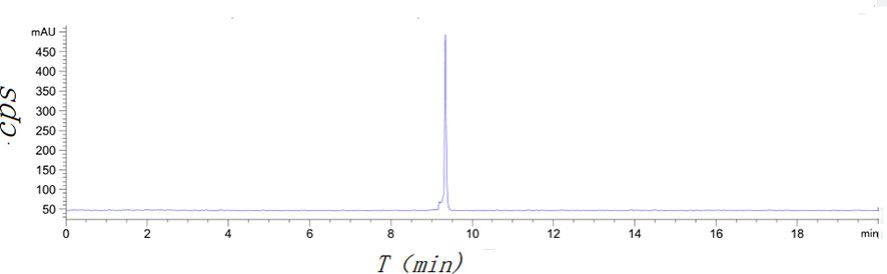
Radiochromatogram of column purified [^18^F]AlF-NOTA-ADH-1.

[^18^F]AlF-NOTA-ADH-1 was successfully prepared using the [^18^F]AlF method (**Scheme 2**). Thin-layer chromatography was performed on the reaction solution. As shown in **Figure 4**, the fluoride ion peak is at the origin, and the Rf of product [^18^F]AlF-NOTA-ADH-1 is 0.5. The non-decay corrected radiochemical yield of [^18^F]AlF-NOTA-ADH-1 was 28.07 ± 2.42% (n=20), the radioactivity of the radiotracer was 310-500mCi, and HPLC analysis showed that the retention time of the drug was 9.4 min. The radio molar activity of the drug was 153.55 ± 28.25 GBq/mmol, and the radiochemical purity was 98.1 ± 0.6% (**Figure 5**). The total time of the radiosynthesis reaction was about 30 minutes. The [^18^F]AlF-NOTA-ADH-1 product was colorless and transparent, with a pH meter of 6.5. The drug is sterile, with bacterial endotoxin levels per 1mL less than 10 EU. The measured partition coefficient of the product was -2.45 ± 0.09, indicating that the probe was hydrophilic. The drug was stable at 37 °C in PBS and calf serum, and the radiochemical purity was about 95.5 and 95.0%, respectively, after 8 h. The results indicated that [^18^F]AlF-NOTA-ADH-1 was stable *in vitro.*

### 3.4 Biological distribution of [^18^F]AlF-NOTA-ADH-1 in normal mice

The research results showed that [^18^F]AlF-NOTA-ADH-1 had rapid blood clearance. After injection of [^18^F]AlF-NOTA-ADH-1, the radioactive concentration of [^18^F]AlF-NOTA-ADH-1 in blood was 18.84 ± 1.95%ID/g at 2 min, 8.77 ± 1.37%ID/g at 5 min, 3.79 ± 1.19%ID/g at 15 min, and 2.28 ± 0.81%ID/g at 30 min, which decreased significantly to 1.04 ± 0.36%ID/g at 120min after injection. [^18^F]AlF-NOTA-ADH-1 is widely distributed in the kidney and the intestines, indicating that [^18^F]AlF-NOTA-ADH-1 is mainly excreted from the urinary system and hepatobiliary systems. In addition, lower uptake was observed in the lungs, and muscle tissues and almost no uptake was seen in the brain tissue, suggesting that the probe does not efficiently cross the blood-brain barrier (**Table 1**).

**Table 1:** Biological distribution of [^18^F]AlF-NOTA-ADH-1 in normal mice( $\bar{x}$ ± *SD*, %ID/g).

Organs	2min	5min	15min	30min	60min	120min
Brain	1.05±0.25	0.87±0.30	0.46±0.10	0.39±0.10	0.22±0.05	0.58±0.68
Heart	9.15±0.91	7.54±2.08	3.57±0.82	3.64±0.66	1.19±0.70	1.01±0.15
Lung	6.26±1.29	4.74±1.69	3.05±0.40	2.15±0.27	2.19±0.42	1.62±0.35
Liver	8.01±1.26	7.09±2.03	14.76±1.11	16.14±2.34	21.62±3.53	13.73±2.35
Spleen	3.84±1.47	5.09±1.29	3.08±0.55	2.66±0.22	1.81±0.57	1.24±0.30
Kidney	24.92±1.12	36.52±2.00	22.06±1.43	25.74±1.17	22.20±1.92	15.18±0.96
Stomach	5.63±1.98	7.27±1.89	4.26±0.55	4.78±0.98	2.32±0.56	1.55±0.20
Intestines	5.39±0.84	6.06±1.42	5.73±0.64	6.28±0.59	5.27±1.09	4.18±0.53
Muscle	3.97±0.56	5.91±1.16	2.79±0.68	3.32±1.77	1.08±0.21	1.22±0.70
Bone	5.30±0.94	6.71±0.62	3.72±0.29	3.09±0.26	1.51±0.36	1.38±0.73
Skin	5.60±0.85	5.08±2.74	3.11±1.47	2.59±0.96	1.85±1.48	1.10±0.96
Blood	18.84±1.95	8.77±1.37	3.79±1.19	2.28±0.81	2.15±0.18	1.04±0.36

### 3.5 Biological distribution of [^18^F]AlF-NOTA-ADH-1 in tumor-bearing nude mice

The biological distribution of normal tissues in tumor-bearing nude mice was similar to that of normal mice, with high radioactive uptake in the liver and kidneys (**Tables 2**–**4**). To investigate the tumor uptake of [^18^F]AlF-NOTA-ADH-1 in the pancreatic PDX xenograft model, tumors, blood, and tissue/organs were excised to measure the radioactivity. The highest tumor uptake was observed at 60 min after injection, suggesting that this may be an appropriate time for tumor imaging. In the blocking group, the radioactive uptake of the tumor was 3.75 ± 0.07%ID/g at 60 min p.i, significantly lower than that in the non-blocking inhibition group (*t*=5.304, *p*=0.006). In SW480 tumor-bearing nude mice, the tumor/muscle (T/M) ratio was the highest at 60min (1.91 ± 0.69). In the blocking inhibition experiment, the radioactive uptake of the tumor was 1.07 ± 0.57%ID/g at 60min p.i, lower than that in the non-blocking group (*p*=0.004). In the BxPC3 tumor model, no significant uptake of [^18^F]AlF-NOTA-ADH-1 was observed in the tumor tissue.

**Table 2:** The biodistribution of [^18^F]AlF-NOTA-ADH-1 in PDX xenograft model( $\bar{x}$ ± *SD*, %ID/g.).

Organs	Non-blocking group				Blocking group
	30min	60min	90min	120min	60min
Blood	0.69±0.22	0.40±0.18	0.39±0.11	0.41±0.07	0.35±0.14
Brain	0.62±0.14	0.99±0.47	0.63±0.18	0.51±0.27	0.86±0.21
Heart	2.54±0.97	0.96±0.17	0.95±0.25	0.81±0.28	0.82±0.02
Lung	4.38±1.33	3.41±0.44	3.52±1.76	2.07±0.94	2.37±0.14
Liver	12.28±1.23	11.31±2.30	10.43±1.97	9.20±1.66	10.35±1.15
Spleen	1.81±0.48	1.41±0.24	1.13±0.03	0.86±0.25	1.25±0.20
Kidney	17.75±5.89	15.82±1.17	12.50±0.89	8.52±2.09	11.62±2.40
Stomach	3.23±1.69	3.40±0.12	6.16±1.58	1.60±1.04	2.24±0.16
Intestines	3.82±0.12	2.14±0.04	3.93±2.67	2.53±2.00	2.49±0.33
Muscle	1.41±0.20	1.56±0.07	2.03±0.05	1.18±0.60	1.68±0.06
Bone	1.74±0.81	2.29±0.96	1.65±1.30	0.74±0.35	2.16±0.09
Skin	0.11±0.04	0.95±0.46	1.51±0.02	1.56±0.66	0.54±0.14
Tumor	8.04±1.82	10.76±2.16	7.18±0.53	4.93±1.65	3.75±0.07
T/Blood	10.72± 1.02	19.21± 2.20	13.54± 0.35	10.27±1.36	9.39± 0.10
T/Muscle	5.69±0.72	8.70±2.68	4.15±1.86	3.97±1.56	2.12±0.06

**Table 3:** The biodistribution of [^18^F]AlF-NOTA-ADH-1 in SW480 xenograft model( $\bar{x}$ ± *SD*, %ID/g).

Organs	Non-blocking group				Blocking group
	30min	60min	90min	120min	60min
Blood	0.30±0.15	0.28±0.06	0.08±0.01	0.12±0.04	0.27±0.09
Brain	0.18±0.05	0.23±0.14	0.19±0.05	0.39±0.24	0.20±0.11
Heart	1.09±0.12	0.67±0.15	0.48±0.08	0.55±0.12	0.59±0.07
Lung	2.58±0.28	1.96±0.56	1.55±0.76	2.34±0.37	1.58±0.45
Liver	8.37±0.87	8.31±3.19	9.32±0.78	8.65±1.56	5.32±1.65
Spleen	0.86±0.26	0.82±0.38	0.71±0.23	0.71±0.15	0.88±0.33
Kidney	8.81±1.80	10.27±3.47	5.73±0.80	5.53±0.91	6.54±2.20
Stomach	5.53±2.81	7.07±0.55	3.13±1.66	1.24±0.01	5.19±1.01
Intestines	1.60±0.78	3.84±2.01	1.87±0.71	1.57±0.97	2.53±0.72
Muscle	0.99±0.19	1.99±1.02	0.78±0.30	0.85±0.14	1.59±0.87
Bone	1.24±0.16	1.63±0.39	0.64±0.17	0.86±0.06	0.84±0.21
Skin	1.01±0.56	1.23±0.37	1.03±0.16	0.95±0.15	1.30±0.29
Tumor	1.73±0.11	2.08±1.64	0.96±0.15	0.73±0.67	1.07±0.57
T/Blood	5.77±0.13	7.42±1.01	9.6±0.76	6.08±0.35	3.96±0.33
T/Muscle	1.75±0.47	1.91±0.69	1.23±0.92	0.86±0.23	1.03±0.56

**Table 4:** The biodistribution of [^18^F]AlF-NOTA-ADH-1 in BxPC3 xenograft models( $\bar{x}$ ± *SD*, %ID/g).

Organ	30min	60min	90min	120min
Blood	0.25±0.05	0.18±0.11	0.09±0.06	0.25±0.19
Brain	0.95±0.61	1.71±0.05	0.49±0.02	0.43±0.29
Heart	1.93±0.46	3.83±0.29	1.70±0.28	1.04±0.06
Lung	2.60±0.23	1.95±0.12	1.06±0.14	0.86±0.56
Liver	6.80±1.65	8.53±1.69	6.73±1.20	5.25±1.51
Pancreas	0.23±0.08	0.61±0.09	0.49±0.23	0.65±0.11
Kidney	9.33±0.73	10.24±0.47	9.39±0.75	6.61±0.87
Stomach	3.12±0.36	2.96±0.42	2.14±0.22	1.78±0.19
Intestines	1.52±0.39	3.55±0.29	0.44±0.21	0.52±0.08
Muscle	1.03±0.40	1.05±0.58	0.91±0.08	0.58±0.28
Bone	2.17±0.19	3.45±0.10	1.90±0.11	1.69±0.39
Skin	3.00±0.74	3.31±0.43	1.23±0.14	0.84±0.19
Tumor	0.89±0.12	1.07±0.36	0.60±0.12	0.37±0.07
T/Blood	3.18± 0.08	4.22±0.23	5.45±0.10	1.42±0.13
T/Muscle	0.86±0.21	0.96±0.32	0.66±0.19	0.63±0.23

### 3.6 *In-vivo* micro-PET imaging studies

**Figure 6:**
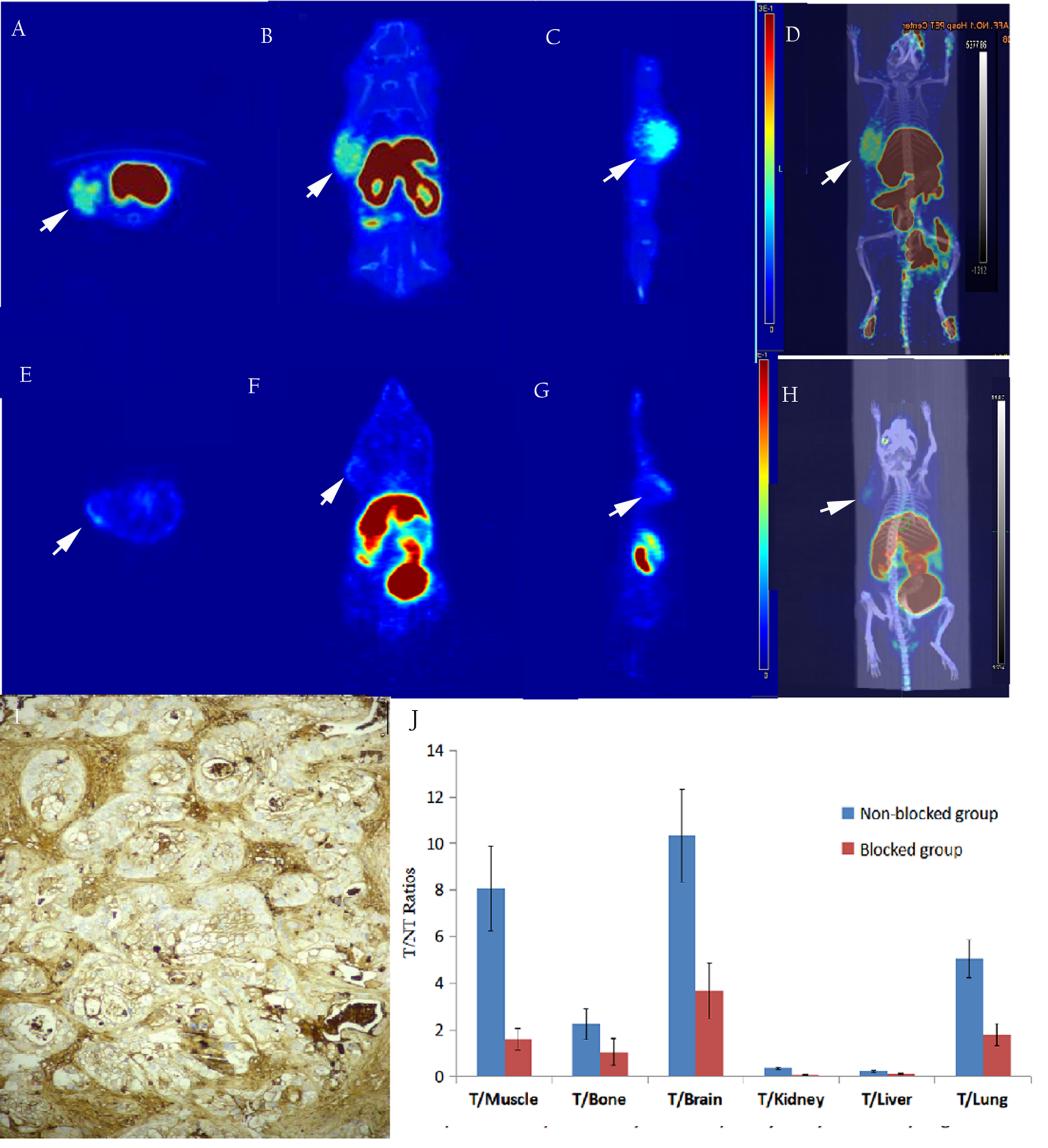
The *in-vivo* micro-PET/CT imaging of subcutaneous pancreatic PDX xenograft model at 60 min after injection of [^18^F]AlF-NOTA-ADH-1, high radioactive uptake of tumor tissue in the unblocked group **(A–D)** and significantly decreased in the blocked group **(E–H)**. Immunohistochemical staining showed a significantly higher expression of N-calcium adhesion (**I**×400); T/NT ratios in the unblocked group and blocked group **(J)**.

Micro PET studies of [^18^F]AlF-NOTA-ADH-1 were performed on nude mice bearing pancreatic PDX, Human Colorectal Carcinoma SW480, and pancreatic BxPC3 tumor xenografts at 60 min p.i. A significantly increased radioactive uptake of tumor tissue was shown in the PDX tumor model, with a tumor/muscle ratio of 8.069±2.832. Unlabeled ADH-1 inhibitors can significantly reduce the T/NT ratios, with a T/Bone of 2.263 ± 0.465, and a T/Lung of 5.062 ± 0.805 (**Figure 6**). The T/NT ratios showed significant differences between the non-blocked group and the blocked group, including T/Muscle (*p*=0.003), T/Bone (*p*=0.023), T/Kidney (*p*=0.0001), T/Liver (*p*=0.002), and T/Lung (*p*=0.001). Immunohistochemical staining showed strong positive N-calcium adhesion expression in tumor tissue, supporting the high specificity of [18F]AlF-NOTA-ADH-1 binding to tumors expressing N-calcium adhesion *in vivo*.

**Figure 7:**
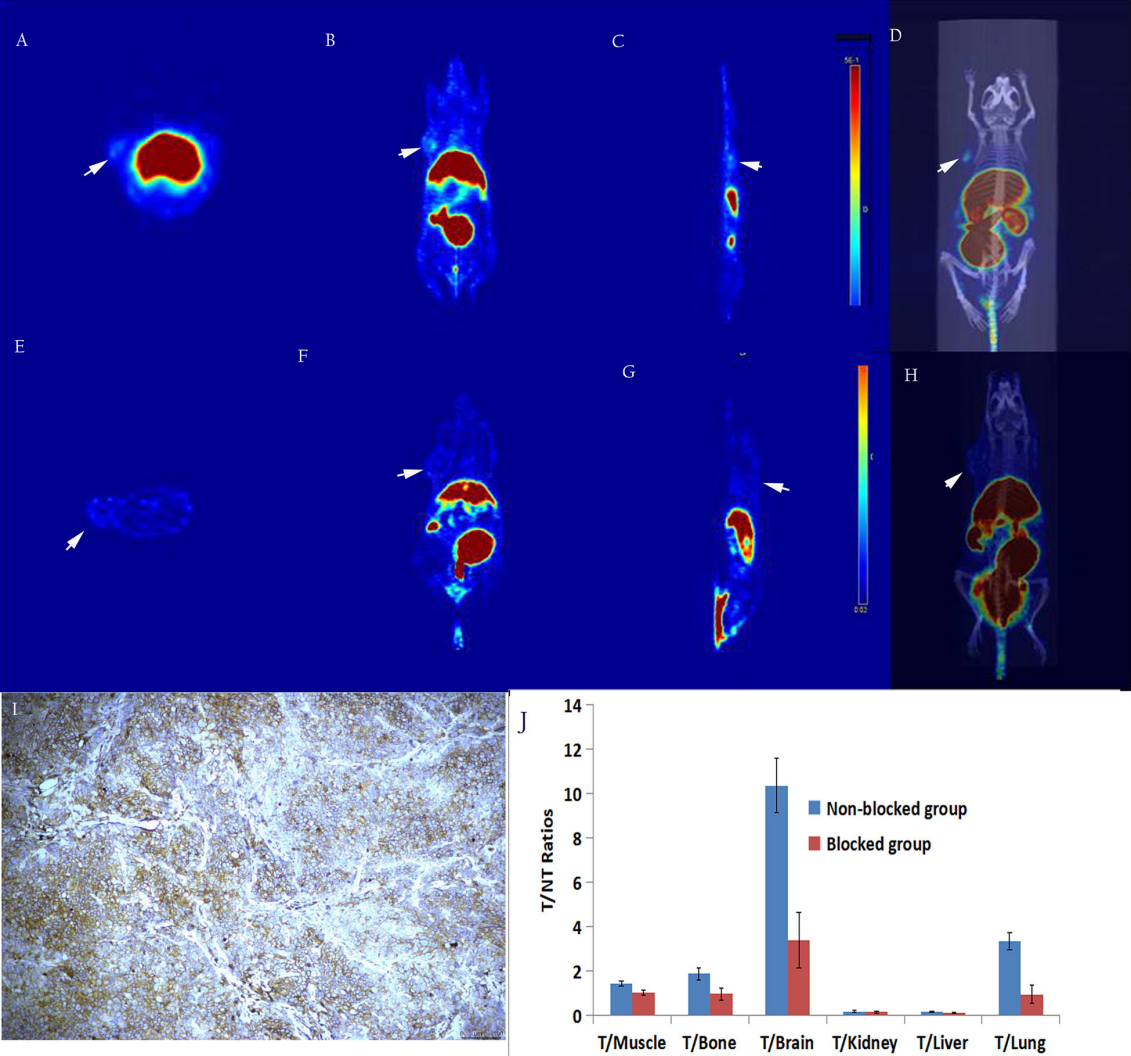
*In vivo* micro-PET/CT imaging of the subcutaneous SW480 xenograft model suggested that the radioactive uptake of the tumor tissue in the unblocked group **(A–D)** was significantly decreased in the blocked group **(E–H)**. Immunohistochemical staining showed a high expression of N-calcium adhesion (**I**,×200); T/NT ratios in the unblocked group and blocked group **(J)**.

**Figure 8:**
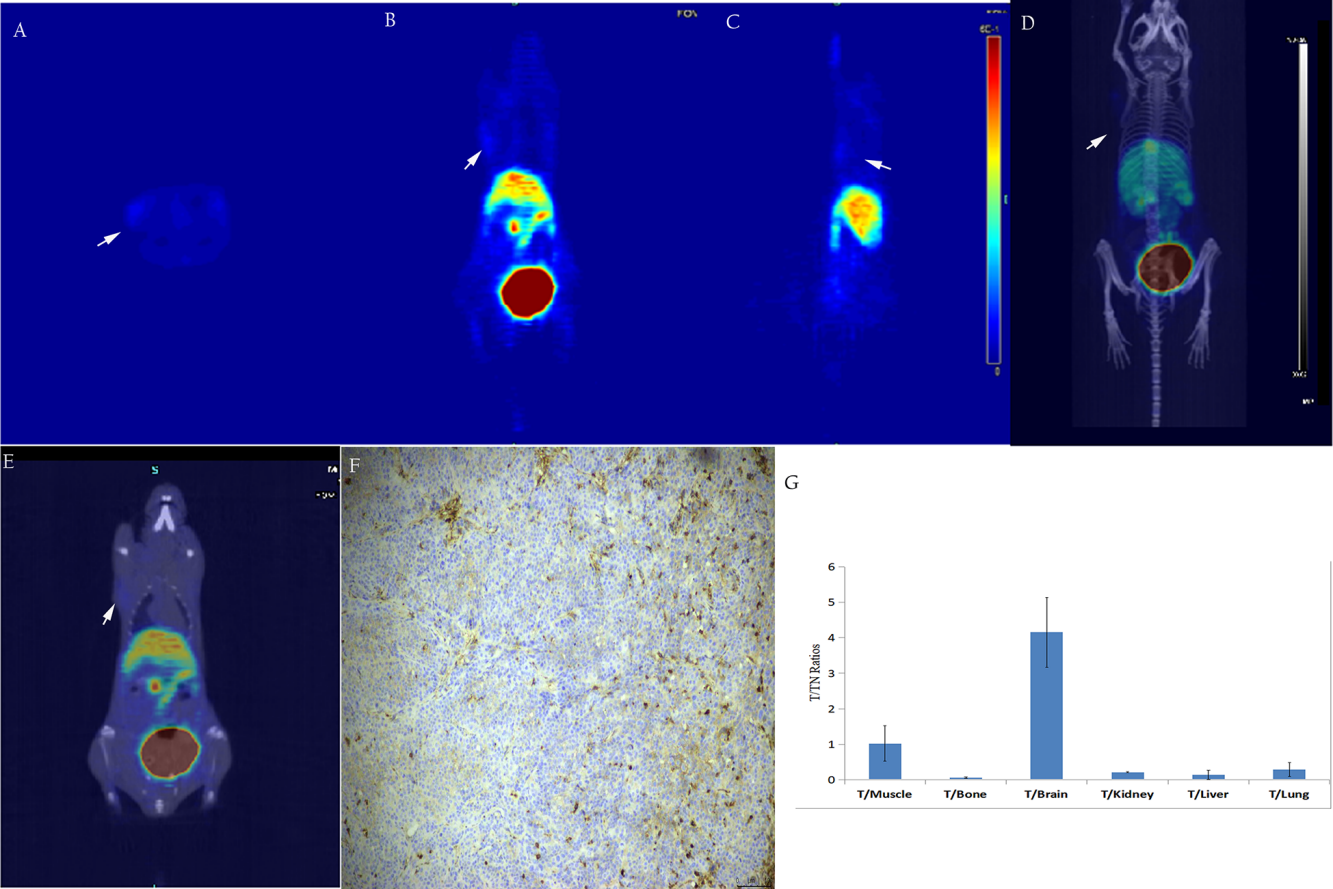
*In vivo*, micro-PET/CT imaging of the subcutaneous BxPC3 xenotransplantation model showed no significant increase in radiation uptake in tumor tissues **(A–E)**. Immunohistochemical staining of N-cadherin in tumor tissue showed low ex-pression in tumor tissue (**F**, ×200); T/NT ratios **(G)**.

As shown in **Figure 7**, micro-PET imaging of the SW480 tumor-bearing nude mouse model 60 min after injecting [^18^F]AlF-NOTA-ADH-1 revealed high radioactive uptake in the tumor with a tumor/muscle ratio of 1.443±0.121, T/Bone of 1.903±0.273, and T/Lung of 3.359±2.998, unlabeled ADH-1 inhibitors can significantly reduce the T/NT ratios, too. A significant difference in T/NT ratios was observed between the non-blocked group and the blocked group, including T/Muscle (*p*=0.019), T/Bone (*p*=0.002), T/Liver (*p*=0.001), and T/Lung (*p*=0.008). Immunohistochemical staining showed positive N-calcium adhesion expression in the tumor tissue. However, no significant increase in radioactive uptake was observed in the tumor tissues of BxPC3-bearing tumor model mice, with a tumor/muscle ratio of 1.018 ± 0.498, T/Bone 0.055 ± 0.022, and T/lung 0.291 ± 0.195. Immunohistochemical staining of N-cadherin showed low expression in tumor tissues (**Figure 8**).

## 4 Discussion

Recent studies have found that the loss of E-cadherin expression in some tumors is often accompanied by the up-regulation of N-cadherin expression (32, 33), which is also known as “the cadherin switch” (33, 34). N-cadherin molecule, also known as neural cadherin, has a molecular weight of 140KD and is mainly expressed in mesenchymal cells, endothelial cells, muscle cells, and hematopoietic stem cells (11). However, recent studies have investigated the up-regulation of N-cadherin in a variety of tumors and its correlation with enhanced tumor invasiveness. In breast cancer, N-cadherin interacts with fibroblast growth factor receptor (FGFR) (35), activates signal pathways, and induces the expression of matrix metalloproteinase-9 (MMP-9) (36), thereby promoting tumor invasion and metastasis (20, 35). In bladder cancer, malignant melanoma, and prostate cancer, N-cadherin promotes tumor cell invasion by activating signaling pathways and inhibits tumor cell apoptosis, which is conducive to tumor cell survival (37, 38). These studies suggest that N-cadherin can not only directly induce cell migration but also promote the survival of tumor cells and facilitate tumor cell invasion. In addition, studies have also shown that N-cadherin-mediated adhesion between tumor cells and mesenchymal cells plays an important role in the process of tumor dissemination and metastasis (34). For example, in the invasive growth of melanoma (39), N-cadherin mediates the interaction between tumor cells and other cells in the dermis. It promotes fibroblasts to form extracellular matrix scaffolds and secrete cytokines and proteases by a paracrine mechanism (40). N-cadherin also plays an essential role in angiogenesis and maturation. Blocking the function of N-cadherin by inhibitory antibodies during embryonic development led to the defect of pericyte recruitment and interfered with angiogenesis (41).

Therefore, N-cadherin can promote tumor invasion and metastasis by inducing cell migration, inhibiting apoptosis, mediating adhesion between tumor cells and mesenchymal cells, and promoting angiogenesis (22). Therefore, N-cadherin could be used as a new target for tumor diagnosis and treatment. ADH-1 is the main N-cadherin antagonist, which significantly alters N-cadherin adhesion, proliferation, and migration, as well as enhancing cell apoptosis in many tumors (11, 18, 42). The phase 1 clinical study involving sixteen human patients with metastatic melanoma, including six patients who had not responded to melphalan alone, revealed that treatment with ADH-1 plus melphalan was effective. Within three months of treatment, eight patients showed a complete response, two had a partial response, two had stable disease, and four had progressive disease (23). Another phase I clinical study showed that the stable period of two patients with ovarian cancer was prolonged (17). However, as a targeted tumor therapy, ADH-1 actually benefits a small number of patients due to the biological heterogeneity of tumors. PET imaging can be used to identify tumor types, screen the patient population that could benefit from targeted therapy, evaluate the treatment response, and predict the treatment effect. In this study, the [^18^F]AlF-labeled ADH-1 probe was radiosynthesized for N-cadherin specific-targeting imaging.

Cy3-labeled ADH-1 and the labeled precursor NOTA-ADH-1 were synthesized by the FMOC method, and the immunofluorescence (IF) staining technique was used to research the cellular uptake of compounds as well as the inhibition test. In addition, Cy3-ADH-1 could bind to SW480 cells at 5 μM, and orange fluorescence was detected at the membrane and in the cytoplasm. The quantity and intensity of tumor cell uptake increased with increasing concentration of Cy3-ADH-1 and reached the highest peak at 50 μM, and then remained constant with the increase of concentration. In contrast, the blank control tube showed no orange fluorescence. Simultaneous inhibition assays showed that SW480 cells were significantly blocked by excess unlabeled ADH-1. The inhibitory effect of 200 nM was better than that of 50 nM excess unlabeled ADH-1. This is in accordance with the receptor-ligand competition binding law. However, the pancreatic cancer BxPC3 cells showed no significant increase in fluorescence uptake in tumor tissues at different concentration intervals from 5 μM to 200 μM, presumably due to the low N-cadherin expression in pancreatic cancer BxPC3 cells, which was confirmed by *in vivo* N-cadherin immunohistochemical staining in nude mice.

^18^F was produced by cyclotron acceleration with a high yield (more than 1000mci), and a half-life is 110 minutes. Compared with ^68^Ga, ^18^F can meet the inspection needs of a large number of patients; it is the ideal positron nuclide for labeling polypeptides (43). In this study, the [^18^F]AlF labeling method was adopted (44, 45). In the incorporation reaction of ^18^FAl and NOTA-ADH-1, the molar ratio of precursor to AlCl_3_ is crucial. The labeling yield was the highest (55%) at an AlCl_3_ concentration of 26 nmol and 5.5 times precursor. However, excessive AlCl_3_ results in decreased labeling yields as Al can form a stable complex with NOTA, competing with ^18^FAl chelation, resulting in a lower labeling rate. Compared with the C-18 column, the yield with the HLB column was higher, and ^18^F ions with high polarity and polar buffer solution could be removed. The radiochemical purity of the obtained product was very high, requiring no further HPLC purification to meet clinical needs. Furthermore, the labeled product was stable in serum, and *in vivo* biodistribution experiments confirmed that the bone uptake was not high, indicating no obvious *in vivo* defluorination.

In order to study the targeting effect of the probe, mice bearing tumors with different expressions of N-cadherin were selected for the study. The biodistribution showed that the probe largely accumulated in the kidneys and livers and subsequently attenuated over time, suggesting excretion mainly through the renal urinary and hepatobiliary systems. The biodistribution results of [^18^F]AlF-NOTA-ADH-1 in the mice bearing pancreatic cancer PDX tumor showed that the probe accumulated more prominently in the tumor, with the highest tumor/non-tumor tissue ratio at 60 min, which was related to the high expression of N-cadherin. The biodistribution in SW480 tumor-bearing nude mice revealed mild uptake of the probe, with the peak tumor/muscle ratio at 60 min, which gradually decreased with time. The mild uptake was also correlated with the moderate staining of N-cadherin expression in the immunohistochemical staining of pathological tissues. In pancreatic cancer BxPC3 tumor-bearing nude mice, the tumor/muscle ratio was lowest, which was consistent with the low expression of N-cad in this tumor type. The biodistribution in different tumors illustrates that the probe is specifically associated with N-cadherin expression in tumor tissue.

In addition, in order to study whether there is competitive inhibition in the tumor tissue, 20 mg/kg of unlabeled ADH-1 solution was coinjected as the block group in the PDX tumor, and the radioactive uptake value of the tumor was significantly lower than that of the non-blocking inhibition group, which was 3.75 ± 0.07% ID/g and 10.76 ± 2.16% ID/g 60 minutes after injection, respectively (*p*=0.006). In the SW480 tumor, the radioactive uptake value was also lower than in the non-blocking inhibition group. Blocking experiments in PDX and SW480 tumor models indicated that [^18^F]AlF-NOTA-ADH-1 has good targeting specificity.

Micro PET/CT imaging of the pancreatic cancer PDX model showed good tumor uptake 60 minutes after injection, with the tumor/muscle ratio reaching 8.70 ± 2.68. Moreover, PDX tumor-blocking experiments showed that tumors could be blocked by a nonradioactive ADH-1 small peptide and attenuated by imaging, which concurred with the biodistribution experiments in the PDX model. The nude mouse model of SW480 demonstrated mild tumor uptake, and SW480 tumors could also be blocked by a nonradioactive ADH-1 small peptide and attenuated by imaging. PET imaging of the BxPC3 pancreatic cancer models showed minimal tumor uptake, and immunohistochemical experiments showed strongly positive N-calcium adhesion expression in PDX tumors, positive expression in SW480 tumors, and low expression in BxPC3 tumors. The micro-PET results showed that the probe [^18^F]AlF-NOTA-ADH-1 could discern the expression of N-cadherin in tumors.

## 5 Conclusions

In the present study, the N-cadherin-targeted fluorescent and NOTA-modified derivatives Cy3-ADH-1 and NOTA-ADH-1 were successfully synthesized. The N-cadherin-targeted PET imaging probe [^18^F]AlF-NOTA-ADH-1 was successfully radiosynthesized. Fluorescence imaging showed that Cy3-ADH-1 could be absorbed by SW480 cells, and its uptake was inhibited by excessive ADH-1, whereas pancreatic cancer BxPC3 cells showed no significant uptake of the fluorescent probe Cy3-ADH-1. *In vivo* biodistribution studies and micro-PET/CT imaging studies of ^18^F-AlF-NOTA-ADH-1 confirmed that the PET imaging probe could distinguish between tumors with different expressions of N-cadherin. Blocking experiments showed that tumors could be blocked by a nonradioactive ADH-1 peptide in PDX and SW480 tumors, suggesting that [^18^F]AlF-NOTA-ADH-1 has the specificity of N-cadherin and the probe had the potential for clinical transformation.

## Data availability statement

The original contributions presented in the study are included in the article/**Supplementary Material**. Further inquiries can be directed to the corresponding authors.

## Ethics statement

The studies involving human participants were reviewed and approved by ethics committee of the First Affiliated Hospital, College of Medicine, Zhejiang University. The patients/participants provided their written informed consent to participate in this study. The animal study was reviewed and approved by Animal Experimental Ethics Committee of the first affiliated hospital, College of Medicine, Zhejiang University.

## Author contributions

M-JD and HL designed the study. Z-FL and Y-QH radiosynthesized the probes and performed Micro-PET imaging. G-HW did cell study in vitro. YD, ZW performed data collection. AA, S-YZ, and G-LW analyzed the results. Q-NY and HZ drafted the manuscript. All authors contributed to the article and approved the submitted version.
