# Supplementary material for: [18F]AlF-NOTA-ADH-1: A new PET molecular radiotracer for imaging of N-cadherin-positive tumors
Source: Front Oncol. 2023 May 22;13:1126721. doi: 10.3389/fonc.2023.1126721 (PMC10239968; doi:10.3389/fonc.2023.1126721)
Supplement: Supplementary file 1 [file DataSheet_1.docx]

**Supporting materials**

**1. Identification of Cy3-ADH-1**

The synthesized Cy3-ADH-1 is a pink powder with a purity of 95.2% by HPLC analysis. The molecular weight of the main peak identified by mass spectrometry analysis is 1228.5（Fig S 1）, in accordance with the theoretical molecular weight of Cy3-ADH-1, which is 1227.5, indicating that the synthesis is correct.

Fig S 1. Mass chromatogram of CY3-ADH-1.

2. Precursor NOTA-ADH-1 synthesis

Through the solid-phase synthesis method, the synthesized NOTA-ADH-1 is a powdery yellow substance. The purity is 98.2% by HPLC analysis, and the main peak molecular weight is 899.5 by mass spectrometry analysis, which is consistent with the theoretical molecular weight of NOTA-ADH-1 of 899.05（Fig S 2）.

Fig S2 Mass chromatogram of NOTA-ADH-1.
